# Submillimeter diameter rotary-pullback fiber-optic endoscope for narrowband red-green-blue reflectance, optical coherence tomography, and autofluorescence *in vivo* imaging

**DOI:** 10.1117/1.JBO.25.3.032005

**Published:** 2019-10-24

**Authors:** Andrea Louise Buenconsejo, Geoffrey Hohert, Max Manning, Elham Abouei, Reid Tingley, Ian Janzen, Jessica McAlpine, Dianne Miller, Anthony Lee, Pierre Lane, Calum MacAulay

**Affiliations:** a British Columbia Cancer Research Center, Department of Integrative Oncology, Vancouver, British Columbia, Canada; b Vancouver General Hospital, Division of Gynecologic Oncology, Vancouver, British Columbia, Canada

**Keywords:** endomicroscopy, fiber optics, red-green-blue imaging, autofluorescence, optical coherence tomography, multimodal imaging

## Abstract

A fiber-based endoscopic imaging system combining narrowband red-green-blue (RGB) reflectance with optical coherence tomography (OCT) and autofluorescence imaging (AFI) has been developed. The system uses a submillimeter diameter rotary-pullback double-clad fiber imaging catheter for sample illumination and detection. The imaging capabilities of each modality are presented and demonstrated with images of a multicolored card, fingerprints, and tongue mucosa. Broadband imaging, which was done to compare with narrowband sources, revealed better contrast but worse color consistency compared with narrowband RGB reflectance. The measured resolution of the endoscopic system is 25  μm in both the rotary direction and the pullback direction. OCT can be performed simultaneously with either narrowband RGB reflectance imaging or AFI.

## 1 Introduction

White light endoscopy is one of the primary diagnostic assessment methods for luminal organs such as the gastrointestinal tract and the respiratory tract. Traditional fiber-optic endoscopes use incoherent fiber bundles for diffuse white light illumination and coherent fiber bundles (CFB) to carry detection light from the distal end to the proximal end of the endoscope.^1^^–^^3^

Endoscopes can employ additional imaging modalities to improve upon conventional endoscopy, which can miss epithelial transformations associated with increased potential of progression to cancer (such as the development of mild, moderate, or severe dysplasia) or carcinoma *in situ*. Narrowband imaging (NBI) is becoming increasingly common in endoscopic imaging and uses specific wavelength ranges of light to enhance the contrast between vasculature and mucosa: blue light enhances vessels in the superficial layers of tissue, whereas green light, with its higher penetration depth, emphasizes deeper vessels.^4^^–^^7^ NBI in conjunction with high-resolution endoscopy (HRE) can improve the differentiation between normal and abnormal tissue based on the visualization of vascular changes in the mucosa,^8^ which is essential in early stage cancer detection. Optical coherence tomography (OCT) provides submillimeter structural information that allows for the differentiation between normal tissue and cancerous or at-risk tissue,^9^ typically with a resolution of a few micrometers,^10^ and has a tissue penetration depth of up to 2.5 mm. The development of endoscopic OCT scanners and catheters has allowed for the imaging of the luminal surface of internal organs.^11^ Autofluorescence imaging (AFI) illuminates tissues with short-wavelength light to excite *in situ* fluorophores, resulting in the emission of longer wavelength fluorescence signals. Potential sites of cancer are then identified by detecting alterations in autofluorescence intensity caused by epidermal thickening and changes to fluorophores and fluorophore concentrations in the submucosa.^12^^,^^13^

The diameter of many commercially available endoscopes is greater than 4.9 mm, making it difficult or impossible to navigate through small-diameter luminal organs such as the endocervical canal, fallopian tubes, and the peripheral lung. For these sites, a miniature, submillimeter endoscope is desired. Large CFB-based endoscopes produce a honeycomb pattern in their images due to the space between fiber cores, often requiring corrections for the missing image information.^14^ This issue is exacerbated in smaller endoscopes that contain fewer imaging fibers. Although CFBs can be replaced by charge-coupled devices (CCD) at the distal end of the endoscope, this approach does not scale down well to submillimeter sizes.^15^ Generally, reducing the diameter of the endoscope reduces the number of resolvable pixels available, thereby reducing the spatial resolution or the field of view (FOV).

Magnification endoscopes that can be used to discriminate features 10 to 71  μm in size have been reported.^16^ In addition, high-definition chip-based endoscopes with pixel counts from about 850k to 2000k are also now available.^17^ However, neither of these have insertion tubes that are submillimeter in diameter due to the magnification lenses and CCD chips needed for these endoscopes.

The optical properties of double-clad fibers (DCF) provide the option to use a single optical fiber in an endoscope, eliminating the size and imaging acquisition issues of CFBs.^18^ DCF-based imaging systems simultaneously carry illumination through one path (the core) and detection light through a second path (the inner cladding).^19^

When connected to a double-clad fiber coupler (DCFC),^20^ which consists of a DCF fused to a multimode fiber (MMF), light traveling in the core can be effectively separated from light travelling in the inner cladding, enabling the combination of multiple imaging modalities such as endoscopic OCT with AFI in a system that makes use of rotational scanning,^21^^,^^22^ or combining OCT with hyperspectral imaging in a system that uses galvo-based raster scanning.^23^

In this work, we present a fiber-based endoscopic system incorporating red-green-blue (RGB) laser illumination for narrowband reflectance in combination with OCT and AFI. This imaging system uses many of the same components and mechanisms from our previous work on a DCF-based OCT-AFI system, where we have used a rotary pullback, DCF-based imaging catheter to capture co-registered OCT and AFI images of pulmonary vascular networks in lung airways.^21^^,^^22^ Narrowband RGB reflectance imaging can potentially complement these two imaging modalities by providing wavelength-specific reflectance or emission information from tissues. The presented imaging system maintains the resolution of high-definition magnifying endoscopes and preserves the high pixel counts of high-definition endoscopes, but omits their magnification lenses and CCD chips, enabling a submillimeter endoscope for imaging small lumens. We demonstrate that the system is capable of co-registered narrowband RGB reflectance and AFI as well as co-registered narrowband RGB reflectance and OCT imaging. Broadband RGB imaging was also performed using the same system.

## 2 Materials and Methods

### 2.1 DCF-Based Imaging Catheter

**Fig. 1:**
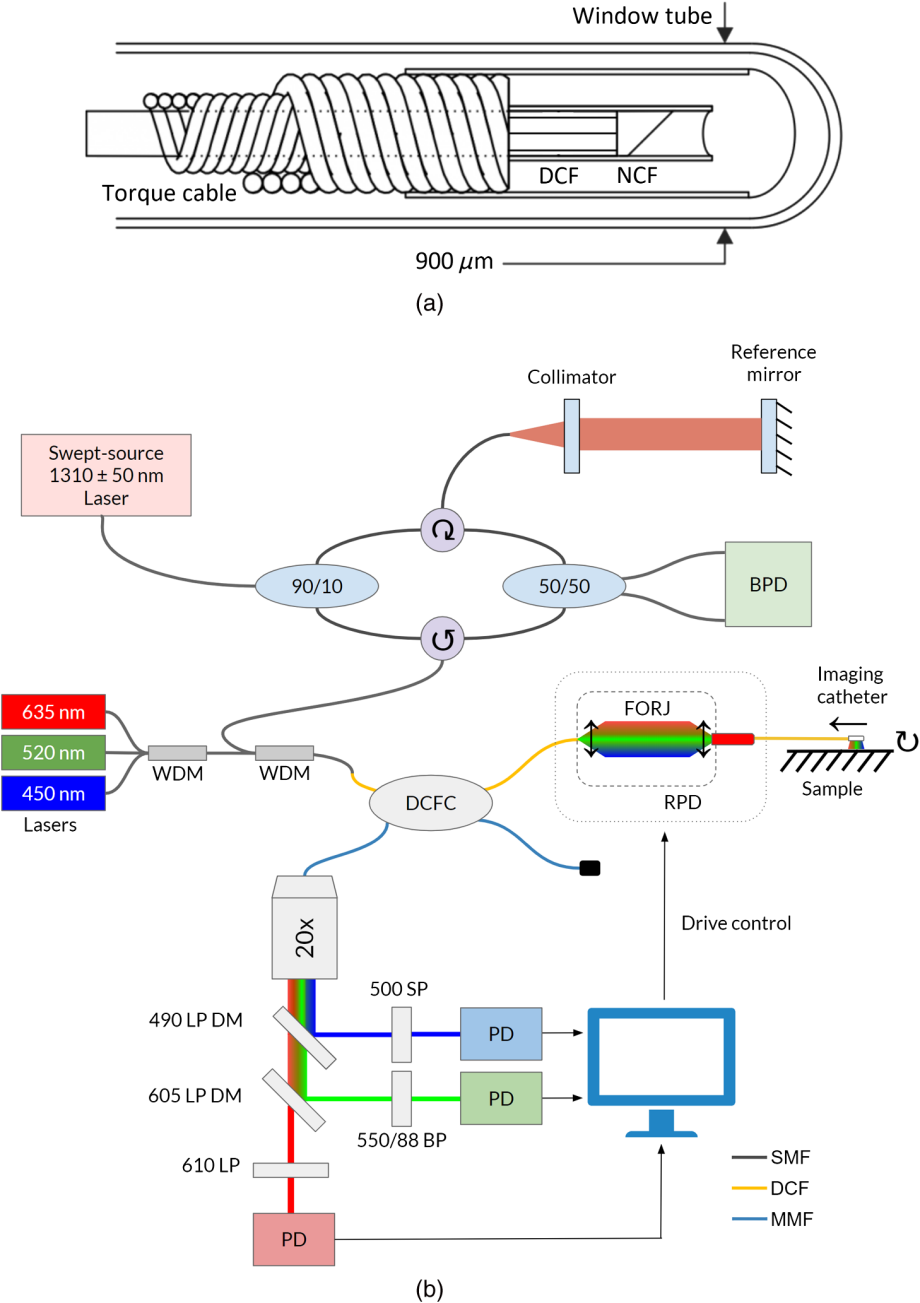
(a) Schematic diagram of DCF-based imaging catheter and (b) the endoscopic system incorporating narrowband RGB reflectance imaging, AFI, and OCT. The imaging catheter consists of an NCF spliced to the DCF and polished at 55 deg relative to the cleave plane. A torque cable is attached for rotary and pullback motion. The assembly is encapsulated within a 900-μm window tube. WDM, wavelength division multiplexer; DCFC, double-clad fiber coupler; FORJ, fiber-optic rotary joint; RPD, rotary-pullback drive; LP, long pass; SP, short pass; BP, bandpass; PD, photodetector; SMF, single-mode fiber; DCF, double-clad fiber; MMF, multimode fiber; and BPD, balanced photodetector.

A schematic of the system used to combine these various modalities is shown in Fig. 1. The custom-made DCF catheter [Fig. 1(a)] consists of a polyimide-coated DCF (9/105/125-20PI, FUD-3489, Nufern, East Granby, Connecticut), in which the core carries the RGB, AFI, and OCT illumination light and the inner cladding returns the AFI and RGB reflectance signals. Scattered OCT light is returned through the core. A no-core fiber (NCF, NCF125, POFC, Hsinchu, Taiwan) is spliced onto the DCF and polished at 55 deg relative to the cleave plane to reflect the diverging imaging beam sideways and slightly forward. The fiber assembly is sealed in a 152-μm outer diameter polyethylene terephthalate (PET) shrink tube to trap air against the polished surface. The entire length is then fixed inside a 500-μm outer diameter double-wound torque cable (Heraeus Medical Components, St. Paul, Minnesota) to transmit rotational and pullback motion from the proximal end to the distal end. A stationary plastic window tube (900-μm outer diameter and 711-μm inner diameter) covers the entire rotating assembly.

The rotational and pullback scanning operation of the DCF-based imaging catheter allows the endoscope to capture 3-D helical scans of tissues. Sampling at a rate of 100 kHz (set to match the laser sweep speed of the infrared OCT laser used as part of the OCT subsystem) allows the system to acquire up to 3072 A-line and reflectance samples per rotation (at a 33-Hz rotational frequency). The imaging catheter can cover an FOV of up to $(5.65\times 140)\;{\mathrm{mm}}^{2}$, achieving a pixel count of ∼8200  k for RGB reflectance, AFI, and *en face* OCT images (using a pullback speed of 0.5  mm/s).

The original OCT-AFI catheters contained an MMF and graded-index (GRIN) fiber spliced to the DCF to improve OCT resolution. As illumination and detection light traveling through common optical elements can frequently introduce unwanted back reflections,^24^ the MMF and GRIN were omitted to decrease the number of index of refraction interfaces and splices within the catheter, thereby reducing the amount of back-reflected light and improving the signal-to-noise ratio (SNR) of the system.

### 2.2 Narrowband RGB Imaging

The imaging system is shown in Fig. 1(b). Fiber-pigtailed laser diodes (Thorlabs Inc., Newton, New Jersey) emitting 450 nm (LP450-SF15), 520 nm (LP520-SF15), and 635 nm (LP635-SF8) light are connected to a wavelength division multiplexer (WDM, RGB26HF, Thorlabs Inc., Newton, New Jersey) to obtain fiber-coupled “white” excitation. A second WDM (WDM6513F, Thorlabs Inc., Newton, New Jersey) combines the RGB light with the infrared light from the OCT subsystem. This light travels through the core of the DCFC (9/105/125, DC1300LEFA, Thorlabs Inc., Newton, New Jersey) and into a custom DCF FORJ (Princetel Inc., Pennington, New Jersey). Custom software controls the rotary-pullback drive (RPD), which spins the catheter and pulls it back at specified speeds from 0.5 to 10  mm/s.

RGB light from the core of the catheter illuminates the sample, and the reflected light is collected by the inner cladding of the catheter, coupled back through the FORJ and into the MMF output of the DCFC. Upon exiting the MMF, the beam is expanded using a 20×, 0.75 NA microscope objective (Fluor, Carl Zeiss Microscopy GmbH, Oberkochen, Germany).

The spatially expanded reflection light is split into red, green, and blue light using a series of filters. These include a 490-nm long-pass dichroic mirror (DMLP490, Thorlabs Inc., Newton, New Jersey), a 500-nm short-pass filter (FES0500, Thorlabs Inc., Newton, New Jersey), a 605-nm long-pass dichroic mirror (DMLP605, Thorlabs Inc., Newton, New Jersey), a 550/88-nm bandpass filter (FF01-550/88-25, Semrock Inc., Rochester, New York), and a 610-nm long-pass filter (610LP RapidEdge, Omega Optical Inc., Brattleboro, Vermont). Three silicon photodiode detectors with switchable gain (PDA100A2, Thorlabs Inc., Newton, New Jersey) are used to capture the individual signals.

### 2.3 Optical Coherence Tomography and Autofluorescence Imaging

The OCT subsystem is a fiber-based Mach–Zehnder interferometer, as described in our previous publications.^13^^–^^14^^,^^21^ Infrared illumination is supplied from a 100-kHz wavelength-swept laser source (SSOCT-1310, Axsun Technologies Inc., Billerica, Massachusetts) centered at 1310 nm and with a bandwidth of 100 nm.

The second WDM (WD6315F, Thorlabs Inc., Newton, New Jersey) splits the OCT reflected signal from the RGB light. OCT reflected light is transmitted through the core of the DCFC and is detected with a balanced photodetector (PDB420A, Thorlabs, Newton, New Jersey).

To perform AFI, the photodetectors in Fig. 1(b) are replaced with photomultiplier tubes (PMTs, H10723-20, Hamamatsu Photonics, Shizuoka, Japan) due to the increased sensitivity needed to detect autofluorescence. Only the blue laser is turned on, and the system captures green and red fluorescence emissions along with blue reflectance simultaneously.

### 2.4 Broadband Imaging

To perform broadband RGB imaging, the laser diodes are replaced by a supercontinuum broadband white-infrared laser source with a wavelength range of ∼430 to 2600 nm (Fianium WhiteLase, NKT Photonics, Birkerød, Denmark). The light is attenuated by a pair of neutral density filter wheels (FW2AND, Thorlabs, Newton, New Jersey) and connected to the illumination port of the DCFC with an SMF-28 patch cord.

The filters in the detection end of the system are replaced to better replicate conventional RGB imaging given the broadband laser source. The 500-nm short pass filter is replaced by a 455-nm SP filter. The 550/88-nm bandpass filter is replaced by a filter with a narrower bandwidth of 535/40  nm (BP 535/40, Delta Optical Thin Film, Hørsholm, Denmark). The 610-nm long-pass filter is replaced by a 630/60-nm bandpass filter (BP 630/60 TP, Delta Optical Thin Film, Hørsholm, Denmark) to reduce the amount of infrared light entering the red photodetector.

### 2.5 Image Processing

The RGB channels were scaled according to the signal from a reflectance-fluorescence target used for white-balancing (uniform reflection of light with respect to wavelength), thereby allowing a normalization of the intensity relationships of the color channels with each other. This information was used for color-balancing the narrowband and broadband RGB images in postprocessing.^25^

As the fiber is not rotationally rigid, rotating the imaging catheter can result in nonuniform rotational distribution (NURD). To reduce the amount of NURD in some of the images, a motion correction method developed by our group denoted “azimuthal en face image registration” was applied to some of the images during postprocessing.^26^

## 3 Results and Discussion

### 3.1 SNR and Resolution

To measure the backscattered signal from a resolution target, the rotating imaging catheter was placed on a flat fluorescence-reflectance ceramic target (Avian Technologies, New London, New Hampshire). The signal from the imaging catheter for a number of rotations without pullback was acquired. The SNR was determined by

$$\mathrm{SNR}=20\;\log_{10}\;\frac{\left\langle I\right\rangle }{\sigma },$$ (1)

where ⟨I⟩ is the difference in intensity between the pixels at the brightest (rotation position pointing directly at target) and the dimmest (rotation position pointing away from the target) rotational locations, averaged across all rotations, and σ is the standard deviation of the pixels at the brightest rotational location across all rotations.

Table 1 shows the SNR of the imaging system for each color channel. The highest SNR was produced by the red channel despite its lower average signal intensity since it displayed the lowest σ. The blue and green channels exhibited comparable values of average intensity, noise, and SNR.

**Table 1:** Signal-to-noise ratio (SNR) values for each color channel.

Color channel	⟨I⟩;	σ	SNR (dB)
Red	6500	207	30.0
Green	13,900	567	27.8
Blue	14,100	567	27.9

**Fig. 2:**
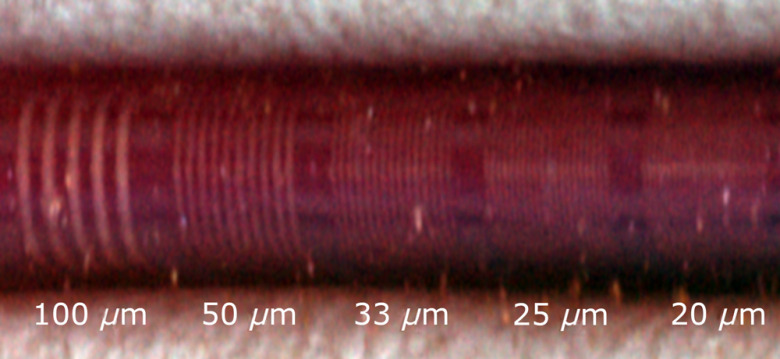
RGB reflectance image of a resolution target indicating fiber-optic resolution in the pullback direction. The resolving power in the rotary and pullback directions is ∼25  μm as the grating frequencies representing these line spacings are still distinguishable.

The resolution of the system was determined using a variable frequency reflectance resolution target (variable frequency target 5 to 120  lp/mm, Edmund Optics, New Jersey). The imaging catheter was oriented on top of the target in both the rotary direction (imaging catheter parallel to the target grating) and in the pullback direction (imaging catheter perpendicular to the target grating), the results of which are shown in Fig. 2. Using narrowband RGB reflectance, the imaging system was able to resolve ∼25  μm in both the rotary direction and the pullback direction. Similar target grating images were recorded for the autofluorescence and OCT imaging modes. These figures fall within the ballpark of expected resolution range of magnification endoscopes.^16^

### 3.2 RGB Reflectance Imaging

**Fig. 3:**
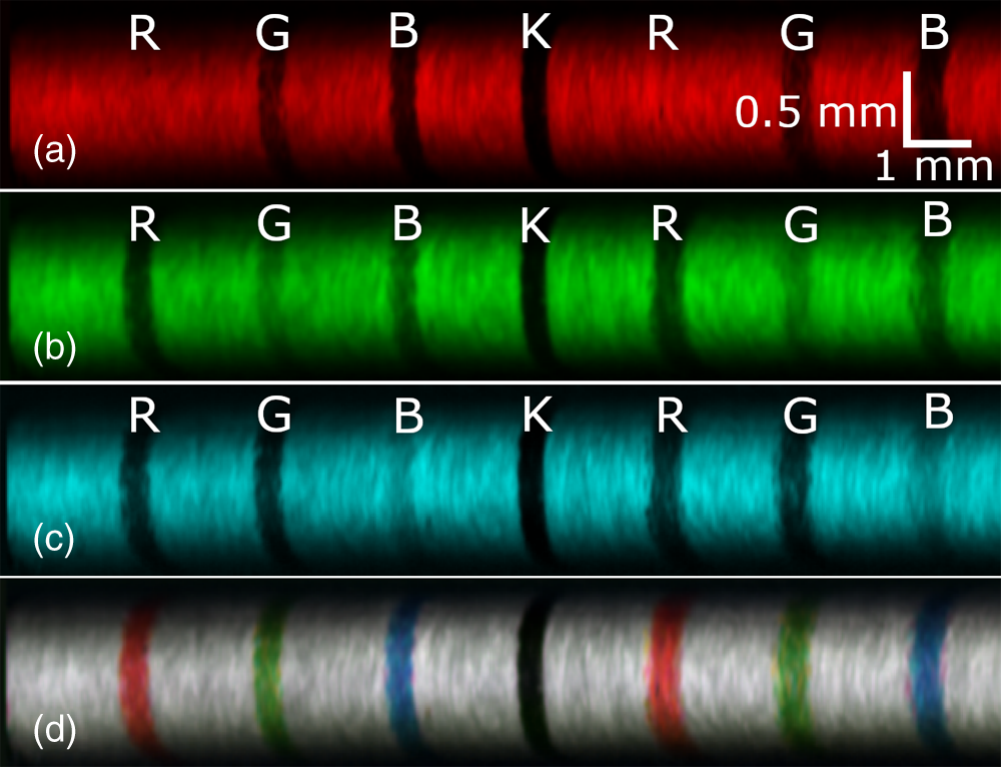
RGB reflectance images of a color card. Printed on the card in a repeated pattern are red (R), green (G), blue (B), and black (K) lines. The fiber, while pressed down on the card, was pulled back perpendicular to the lines. (a)–(c) The false-color images obtained from each color channel and (d) the composite RGB image.

A white paper card printed with red, green, blue, and black (RGBK) lines was imaged to demonstrate the color recognition abilities of the system. The imaging catheter was placed onto the card and a rotary pullback was performed to obtain these images. The grayscale images of the red, green, and blue channels were combined to produce an RGB image of the card (Fig. 3). The resolution of the system is sufficient to visualize the wood fibers making up the paper card.

Figures 3(a)–3(c) show that the light shone on dissimilar colored lines was absorbed more and reflected less, hence the darker appearance of the lines. Even the color lines matching the color of the illumination light are slightly darker due to the partial absorption of the light by the printer ink. Overall, the RGB composite [Fig. 3(d)] compared well with the visual appearance of the color card.

**Fig. 4:**
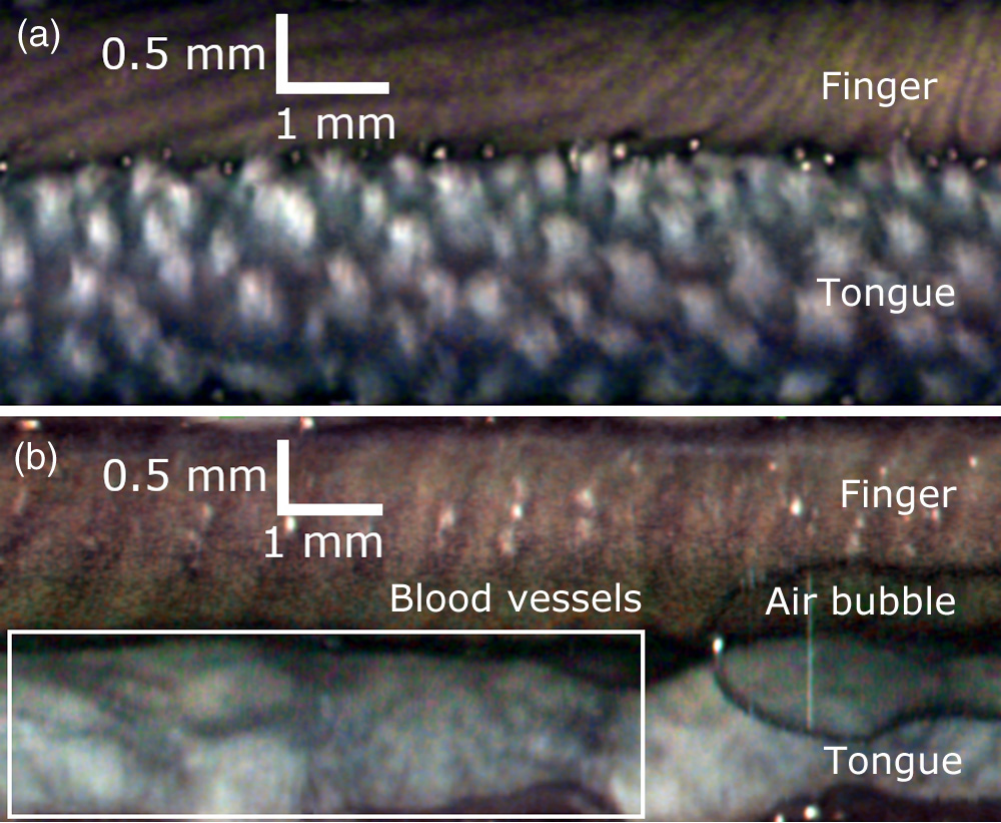
RGB reflectance images of (a) the top of a tongue and (b) the ventral (underside) of the tongue. Fingerprints, tongue papillae, and blood vessel features are captured.

Figure 4(a) shows the RGB reflectance image of a tongue acquired using a finger to press the imaging catheter against the tongue. The most prominent details are the papillae on the tongue, showing that the system can capture surface structure information from tissue. The ventral (underside) of the tongue was similarly imaged to capture blood vessels visible on the surface, which correspond to the dark and slightly blue streaks in Fig. 4(b). This demonstrates the system’s ability to capture surface details of tissue and to visualize vasculature within tissue.

While performing this image acquisition, an air bubble between the PET shrink tube and the window tube was dragged by the imaging catheter during part of the fiber pullback [Fig. 4(b)].

### 3.3 Optical Coherence Tomography and Autofluorescence Images

**Fig. 5:**
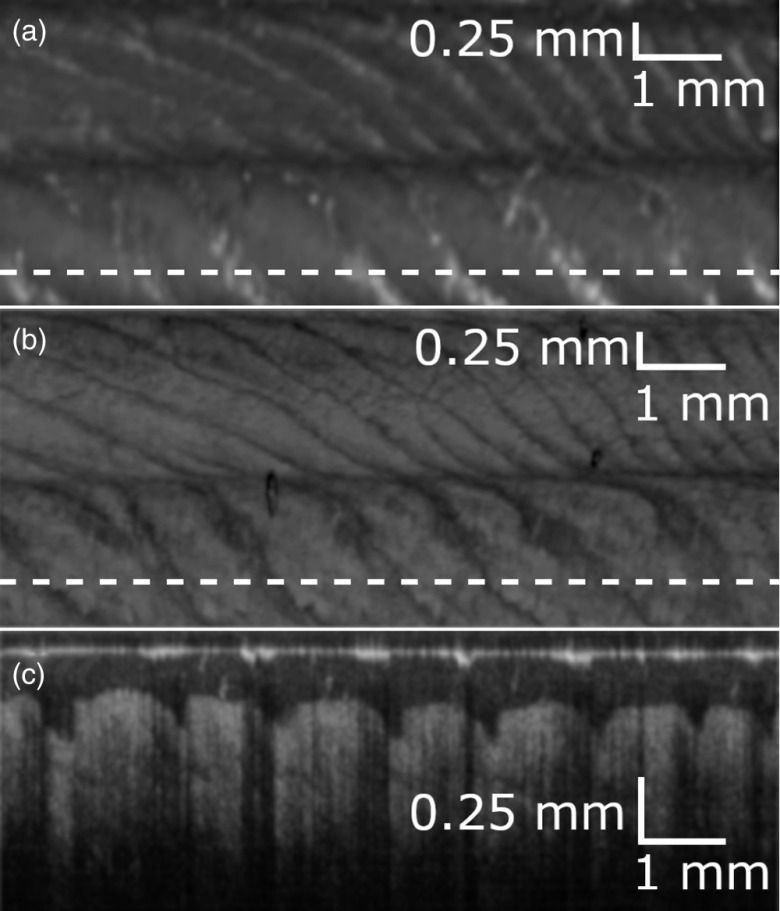
RGB reflectance: (a) grayscale for feature emphasis, (b) *en face* mean intensity projection OCT, and (c) longitudinal OCT co-localized with dashed lines on (a) and (b).

Figure 5 shows simultaneously acquired co-registered RGB reflectance and OCT images. Although the same structures are visible, the details are different between the RGB intensity image [Fig. 5(a), transformed into grayscale for the comparison] and the *en face* OCT image [Fig. 5(b)]. The dashed lines superimposed on images (a) and (b) indicate the rotational angle for which the depth cross section is shown in the longitudinal OCT image in Fig. 5(c).

**Fig. 6:**
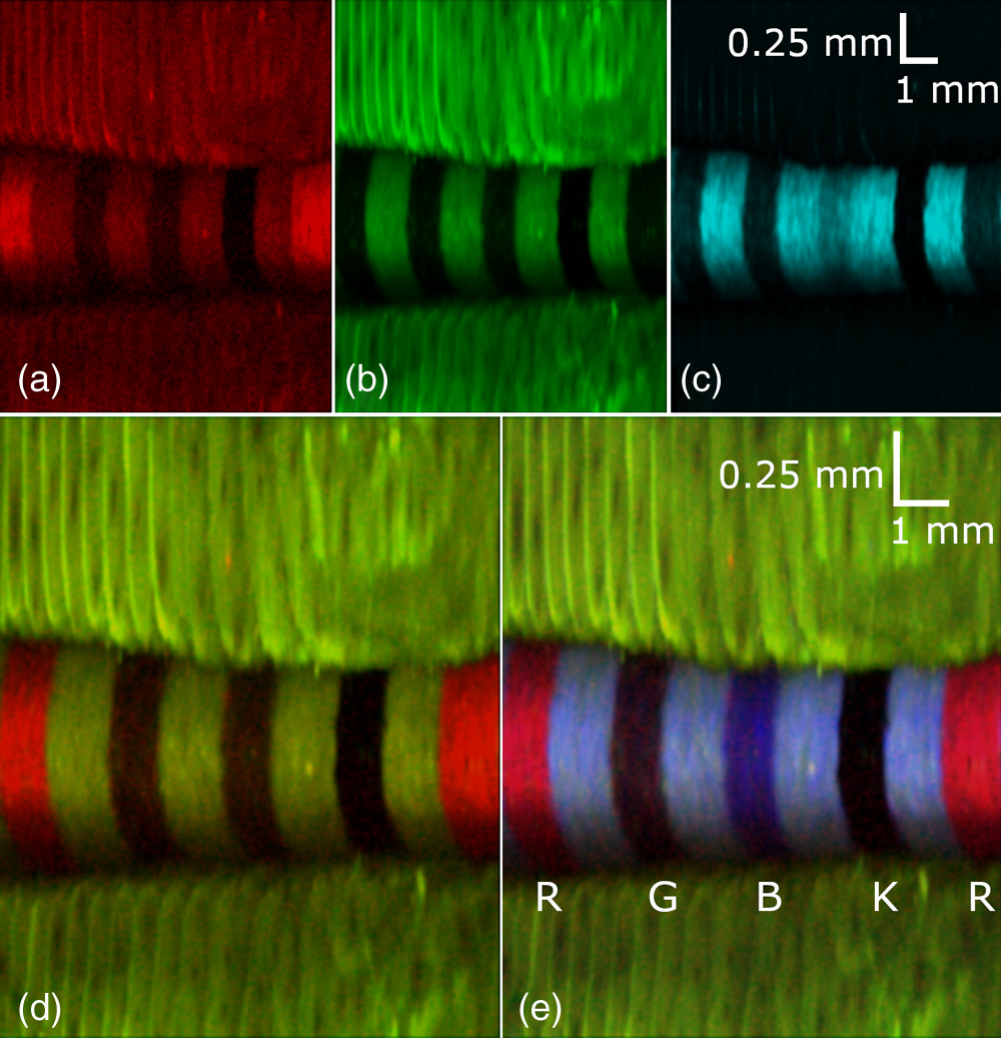
Autofluorescence and reflectance images of the RGBK card taken with photomultiplier tubes: (a) red autofluorescence, (b) green autofluorescence, (c) blue reflectance, (d) co-registered red and green autofluorescence, and (e) co-registered red and green autofluorescence and blue reflectance. It appears in the image that the red ink used to mark the card exhibits strong red autofluorescence when illuminated with blue light.

To perform AFI, only blue excitation light was used to illuminate the sample. Autofluorescence and reflectance images of the RGBK color card were taken with PMTs (Fig. 6).

The red autofluorescence image [Fig. 6(a)] shows that the red lines on the card fluoresce significantly. The green lines in the green autofluorescence image [Fig. 6(b)] of the card do not fluoresce nearly as much. The finger pressing the imaging catheter down onto the card is still visible in both images, but it does not show up in the blue reflectance image [Fig. 6(c)] due to the higher reflectivity of the card compared with that of the finger. Figure 6(d) shows co-registered red and green autofluorescence, whereas Fig. 6(e) shows co-registered autofluorescence from the red and green channels and blue reflectance.

**Fig. 7:**
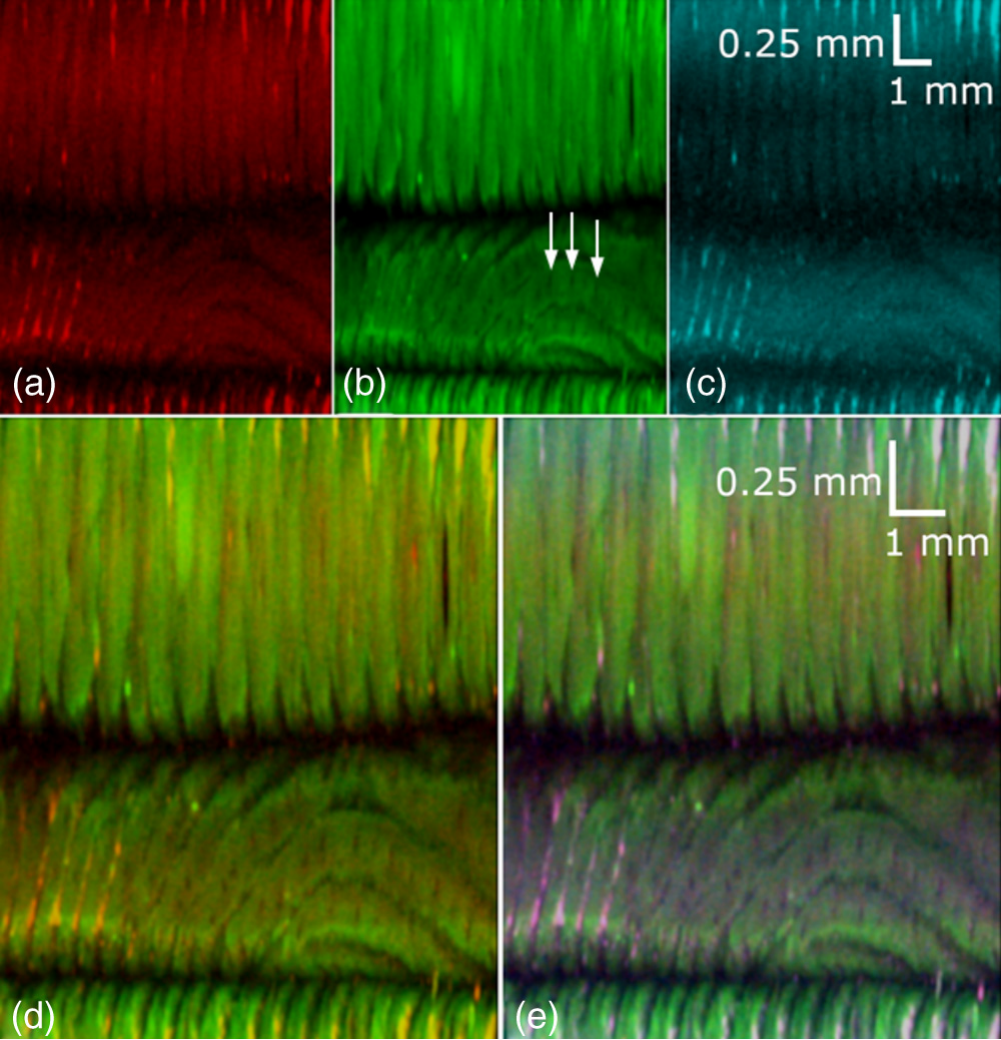
Autofluorescence and reflectance images of fingers taken with photomultiplier tubes: (a) red autofluorescence emission, (b) green autofluorescence emission, (c) blue reflectance, (d) co-registered red and green autofluorescence, and (e) co-registered red and green autofluorescence and blue reflectance. The arrows in (b) indicate sweat pores that run along the ridges of the fingerprint.

The autofluorescence and reflectance images from a finger are shown in Fig. 7. Sweat pores along the fingers are particularly visible in the green autofluorescence image as low-fluorescence spots on the ridges of the fingerprint [Fig. 7(b)]. The fingerprint in the blue reflectance image [Fig. 7(c)] does not have as much contrast as the autofluorescence images due to the amount of surface backscatter obtained together with the signal. The co-registered red and green autofluorescence image is shown in Fig. 7(d), whereas the co-registered red and green autofluorescence and blue reflectance image is shown in Fig. 7(e).

The calibration step performed for RGB reflectance imaging is not performed for the autofluorescence signals. Instead, these signals are contrast stretched such that their lower 1% and upper 99% intensities are scaled to 0 and 255, respectively.

### 3.4 Broadband Images

**Fig. 8:**
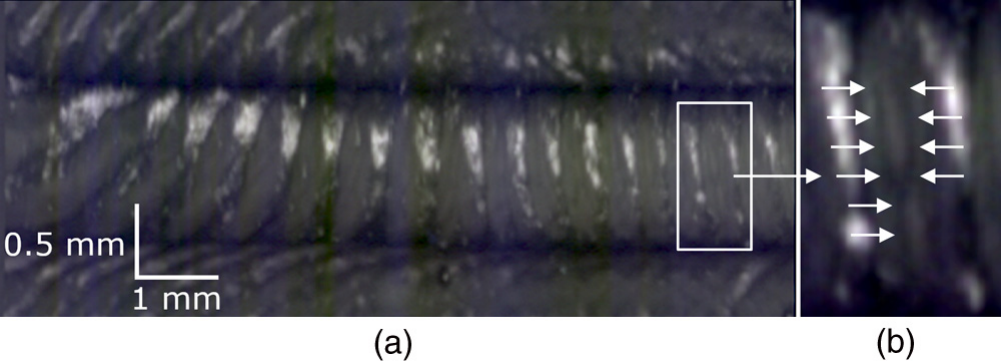
(a) Broadband RGB reflectance image of a finger using a supercontinuum white light source and (b) the contrast-enhanced close-up image further fingerprint structure in the form of striations in between ridges.

Shown in Fig. 8(a) is the broadband RGB image of a finger captured using the supercontinuum white laser light as the illumination source. The system produced images with improved contrast evident in the broadband image of fingerprints compared with its narrowband RGB laser reflectance equivalent (Fig. 4). Striations between fingerprint ridges are now visible, as shown in Fig. 8(b) (contrast-enhanced). These are not visible in the narrowband RGB images of fingers. Vertical green bands present in Fig. 8(a) are artifacts due to wavelength-dependent output fluctuations from the supercontinuum laser.

OCT can be performed at the same time as either RGB reflectance or AF, but since the red and green detectors are shared between reflectance and AFI modes, acquisition of each mode must be done separately. The endoscopic imaging system presented here can further be improved by pulsing the laser diodes to separate the acquisition, RGB, and autofluorescence modes in time, and the three modalities would be able to function simultaneously.

The endoscopic imaging system shows great potential to perform the *in vivo* imaging of intraepithelial lesions within small lumina in various sites, such as the cervix, fallopian tubes, and lungs. Accomplishing this would allow for the early detection and localization of cancers in these sites.

## 4 Conclusions

We have demonstrated a narrow (submillimeter) side-viewing endoscope that allows the color visualization of epithelial surfaces with the resolving power of magnification endoscopy and the high pixel counts of HRE while still being suitable for use in the more distal and smaller luminal diameter internal organ sites. This fiber-optic endomicroscopy system is capable of RGB reflectance, AF, and OCT imaging and is able to resolve details at least 25 μm in size, for surfaces in contact with the probe, while scanning areas ∼3 to 4 mm in circumference over 140-mm in length. Images of fingers, oral cavity (tongue), and blood vessels were presented.
